# Critical Decision Thresholds for Urgent Physician Notification of Point-of-Care Testing Results

**DOI:** 10.3390/diagnostics16081139

**Published:** 2026-04-10

**Authors:** Kami Osher, Gerald J. Kost

**Affiliations:** 1 University Honors Program, University of California, Davis, CA 95616, USA; kami.osher@gmail.com; 2 Pathology and Laboratory Medicine, School of Medicine, University of California, Davis, CA 95616, USA

**Keywords:** critical limits, critical values, decision thresholds, demographics, national database, instruments, point-of-care testing, standards of care, artificial intelligence (AI)

## Abstract

**Background/Objectives**: Critical limits define quantitative thresholds for life-threatening diagnostic test results that require immediate clinician notification and may prompt urgent intervention to prevent adverse outcomes. This study aims to (1) characterize point-of-care (POC) critical limits for adults and newborns using a comprehensive U.S. national database, (2) identify POC instruments associated with these limits, and (3) support harmonization of point-of-care testing (POCT) practices. **Methods**: We gathered critical limit notification lists from 417 hospitals across all 50 states and Washington D.C., comprising university hospitals, trauma and heart centers, centers of excellence, community hospitals, and network hospitals. We extracted POC and central laboratory critical limits (at hospitals with POC), adult international normalized ratio (INR) data, and instrument usage. **Results**: Low and high glucose critical limits were the most frequently listed POC thresholds, with median values of 50 and 450 mg/dL, respectively, reported by 73 hospitals (17.5%). Troponin was listed by ten hospitals, specified as troponin (*n* = 4), troponin I (*n* = 5), or “troponin TnI” (*n* = 1). A few hospitals assigned instrument-specific critical limits for the same analyte, and 55 hospitals did not specify instrument usage for any measurand. Median differences in matched pairs of laboratory versus POC critical limits differed significantly (Wilcoxon signed-rank, *p* < 0.05) for low and high ionized calcium (*n* = 21), low hemoglobin (*n* = 23) and high INR critical limits for adults (*n* = 27) and newborns (*n* = 10). In some cases, matched pair analytes demonstrated identical critical limits. **Conclusions**: Harmonizing critical limit notification thresholds across point-of-care testing and different devices may improve consistency in clinical decision-making and patient outcomes. Despite the potential of POCT to shorten time to urgent intervention, relatively few hospitals currently include POCT critical limits on notification lists. Establishing standards, annual updating, and enforcing risk mitigation could enhance adoption and reliability. Broader inclusion and transparent sharing of POCT critical values could harmonize practices across institutions, facilitate inter-institutional collaboration, and promote more timely and reliable responses to life-threatening diagnostic results.

## 1. Introduction

The urgent communication of life-threatening test results is vital for patient safety, enabling rapid, potentially life-saving interventions. Unlike traditional laboratory testing, point-of-care testing (POCT) delivers rapid diagnostic results directly at or near the patient’s site. Healthcare institutions set thresholds for dangerously abnormal results to guide immediate treatment. The 2026 National Patient Safety Goals (NPSG) of The Joint Commission [1] call for timely notification of life-threatening test results; NPSG.02.03.01 requires documentation of compliance and evaluation of the timeliness of reporting critical test results to clinical providers.

POCT is widely used for its speed and accessibility [2]. Adoption depends on institutional needs and resources, with strict oversight ensuring quality when POCT is employed. However, despite its rapid turnaround and increasing clinical reliance, the criteria used to define critical limits in POCT are not standardized across institutions. The lack of national standards for point-of-care (POC) critical limits leads to variability in testing practices, policies, and documentation. This variability may result in inconsistent clinical responses to similar patient conditions, potentially delaying intervention or introducing avoidable patient risk.

Focused research on the standardization and reporting of critical limits specifically for POCT remains limited. This study reports point-of-care critical limits from a 2025 U.S. national database. We characterize listing frequencies for POC analytes, assess instrument usage, and document inconsistencies in reporting and documentation of POC critical limits. Our work provides new insight into current practices, highlights risk of inconsistencies and identifies opportunities for clearer guidance to improve patient safety.

## 2. Materials and Methods

### 2.1. Definitions

A critical limit is defined as a low or high quantitative threshold of a life-threatening diagnostic test result [3]. A critical value is defined as a qualitative result (such as a positive COVID-19 rapid antigen test) warranting urgent notification. Both demand rapid response and potentially life-saving treatment, isolation of the patient, or other timely medical interventions [4]. Point-of-care testing is medical testing at or near the site of patient care.

### 2.2. National Database

We collected critical limit notification lists from 417 hospitals across all 50 states and Washington D.C., ensuring complete geographic representation. No survey forms were distributed. Raw data in the form of notification lists used by hospitals were gathered from 2023 to 2025.

The national database comprised university hospitals and affiliates, heart centers, trauma centers [total of 238 with 97 Level I (40.8%), 38 Level II (16.0%), 58 Level III (24.4%), and 45 Level IV (18.9%)], centers of excellence (80% of U.S. News and World Reports’ Top 40), and community hospitals. Notification lists reflect posted policies in use by individual and network hospitals. We extracted point-of-care critical limits, central laboratory critical limits (at hospitals with POC), adult international normalized ratios (INR), and instrument usage. Critical values were transferred to Excel spreadsheets and verified to exclude transcription errors.

### 2.3. U.S. Census Geographic Divisions

The national database of critical limit notification lists in use by hospitals in all 9 U.S. census divisions covers 50 states and Washington DC: Division (1) New England, *N* = 17 hospitals (CT, MA, ME, NH, RI, VT); (2) Middle Atlantic, 33 (NJ, NY, PA); (3) East North Central, 69 (IL, IN, MI, OH, WI); (4) West North Central, 75 (IA, KS, MN, MO, ND, NE, SD); (5) South Atlantic, 71 (Wash. DC, DE, FL, GA, MD, NC, SC, VA, WV); (6) East South Central, 25 (AL, KY, MS, TN); (7) West South Central, 30 (AR, LA, OK, TX); (8) Mountain, 47 (AZ, CO, ID, MT, NV, NM, UT, WY); and (9) Pacific, 50 (AK, CA, HI, OR, WA).

### 2.4. Statistics

The order of measurands in the tables was based on the frequencies of the 2025 listings. Listing frequencies were calculated by dividing the number of hospitals reporting each measurand by 417 (the total number of hospitals).

POCT critical limits were not normally distributed. Hence, we applied nonparametric statistical tests. We used the nonparametric Wilcoxon signed-rank test to determine significant differences between POC versus laboratory matched pairs of critical limits at the same hospitals. Please note that with the Wilcoxon signed-rank test, a significant difference may result from the distributions being dissimilar, even when the medians might be the same. Effect sizes (rank-biserial correlations) refer to a standardized measure of the magnitude and direction of the paired difference in the Wilcoxon signed-rank test, ranging from −1 to +1, where values closer to ±1 indicate stronger effects and the sign indicates the direction of change.

JASP (Version 0.96.0) and other open-source (web-based) software were used for statistical computations. JASP is a free statistical software developed with support from the University of Amsterdam, implemented in R, and designed for easy user accessibility [5].

### 2.5. Standard Inclusion/Exclusion Criteria

We excluded courtesy call results and alert values. We included critical limits and values that were identified as point of care, rapid lab, bedside, satellite labs, MICU, and Rapid Response Lab. We analyzed POC instruments identified with critical limits listings, including Nova, i-STAT, Piccolo, Clinitek, Hemocue, Lead Care II, RapidPoint, Accu-Chek, and others.

When several age ranges fell within the same category and listed different values, we took the most conservative values (highest low value and lowest high value). The same applies for notification lists that specify different critical limits for different devices measuring the same analyte. Measurands with a listing frequency of 2% or lower are reported in the Supplementary Materials.

### 2.6. Artificial Intelligence and Web Searches

Open-source artificial intelligence, primarily Perplexity and ChatGPT (version 5.3), and Internet search engines (e.g., Google and PubMed) were employed to accelerate raw data acquisition and literature searches. When available, notification lists were obtained from the Web and verified directly with laboratory directors via email. Artificial intelligence was not used to write this report.

### 2.7. Units

Glucose in mg/dL was converted to mmol/L using the formula: mmol/L = mg/dL × 0.05551. Ionized calcium in mg/dL was converted to mmol/L using the formula: mmol/L = mg/dL × 0.25. Total bilirubin in mg/dL was converted to µmol/L using the formula: µmol/L = mg/dL × 17.1. pO_2_ and pCO_2_ in mmHg were converted to kPa using the formula: kPa = mmHg × 0.133322. We report results in both conventional and Systeme International (SI) units for accessibility by international readers.

### 2.8. Ethics

The UC Davis IRB deemed this research exempt (ID 2078118-1). Data were collated statistically and reported anonymously. No consents were required.

## 3. Results

### 3.1. Number of POC Tests on Notification Lists

**Figure 1:**
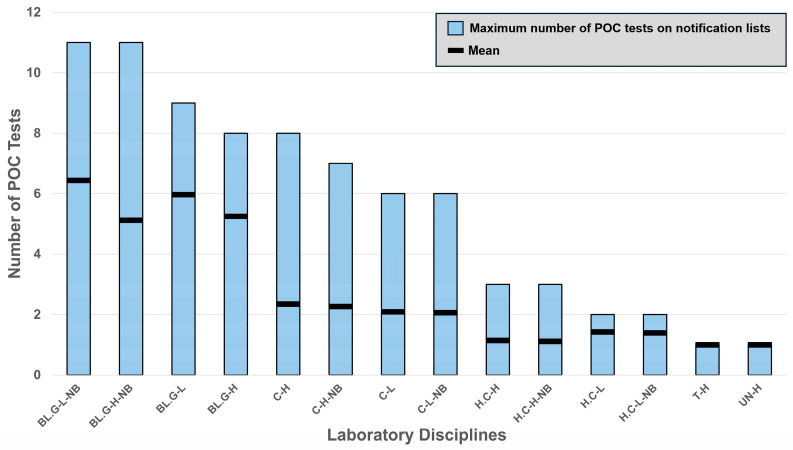
Number of POC tests on notification lists. Bars represent the range in the number of point-of-care tests on notification lists, grouped by laboratory discipline. The x-axis shows pairs of low and high critical limits, with separate groups for newborns and adults for each discipline, where applicable. The horizontal black line within each bar denotes the mean value, excluding zeros. Abbreviations: BL.G, blood gas and pH; C, clinical chemistry; H.C, hematology and coagulation; T, toxicology; UN, urinalysis; NB, newborn; L, low critical limits; and H, high critical limits.

From our national database, only 94 (22.5%) included point-of-care critical limits and values on their notification lists, representing a comprehensive count of hospitals reporting at least one POC analyte. Figure 1 presents a Pareto plot showing the range and average (excluding zero) number of point-of-care tests on notification lists. The total number of POC tests across all disciplines for adults were 16 low (“L”) and 15 high (“H”) critical limits, and for newborns (“NB”) 15 low and 13 high critical limits.

### 3.2. Instruments

**Figure 2:**
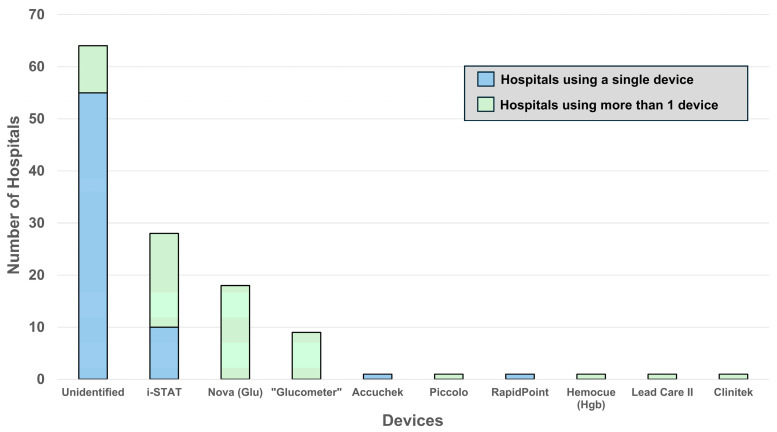
Number of hospitals identifying different POC instruments with critical limit notifications. [“Glucometer” is a trade name.]

Figure 2 presents a Pareto plot showing the number of hospitals using various devices. The figure is color-coded by instrument count—blue indicates hospitals using a single device, and green indicates hospitals using multiple devices. Fifty-five hospitals did not specify instrument usage for any measurand on their notifications lists and nine hospitals specified instrument usage for only some measurands.

### 3.3. Chemistry Tests

Table 1 summarizes the POC clinical chemistry low and high critical limits for adults and newborns (in adult hospitals). Low and high glucose critical limits were the most frequently listed point-of-care tests, reported by 73 hospitals (17.5%) for adults and 62 hospitals (14.9%) for newborns. Only one hospital listed 70 mg/dL (3.9 mmol/L) as the low glucose critical limit. The American Diabetes Association (ADA) defines glucose <54 mg/dL (<3.0 mmol/L) as Level 2 hypoglycemia and from 54 to <70 mg/dL (<3.9 mmol/L) as Level 1 hypoglycemia. [6,7]. Ten hospitals (2.4%) listed cardiac biomarkers comprising troponin (four hospitals), troponin I (five), or “troponin TnI” (1). Five identified the instrument used, i-STAT.

**Figure 3:**
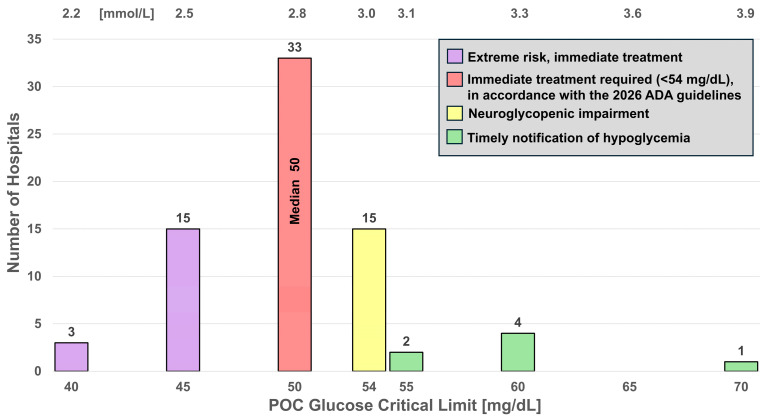
Histogram for low critical limits for POC glucose. Colors identify risk zones, with purple indicating extreme risk. Critical limits within the red zone require immediate treatment (<54 mg/dL) in accordance with the 2026 ADA guidelines. The green zone can be considered safer, but patients should still be evaluated for neuroglycopenic impaired awareness. For assignment of risk levels, please see the Discussion and the references cited therein.

**Figure 4:**
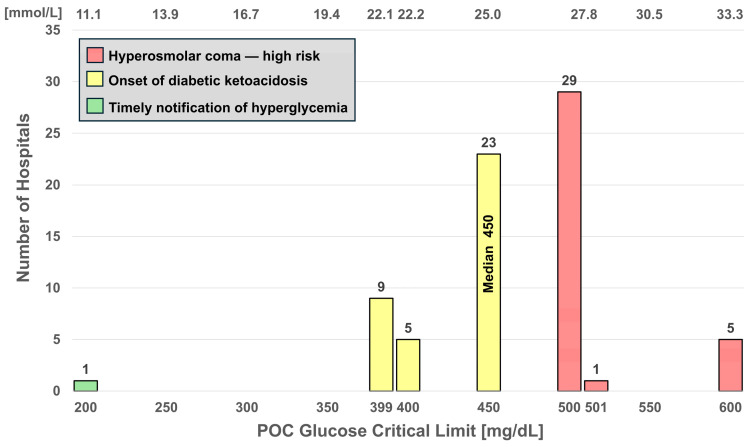
Histogram for high critical limits for POC glucose. Colors identify risk zones, with red indicating extreme hyperglycemia.

Figure 3 and Figure 4 display histograms for low and high glucose critical limits, respectively. Figure 3 displays a wide distribution of low critical limits with a range of 40–70 and a median of 50 mg/dL and is color-coded for risk zones (green, low risk; yellow, medium; red, high; and purple, extremely high). In Figure 4, color-coding reflects timely notification of hyperglycemia (green), onset of diabetic ketoacidosis (yellow), and increasing risk of hyperosmolar coma (red). For the glucose high critical limits, the range is 200–600 mg/dL, and the median 450 mg/dL.

**Figure 5:**
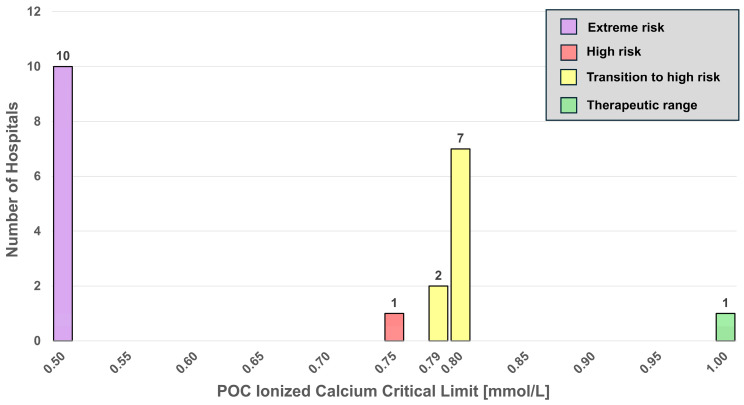
Histogram for low critical limits for ionized calcium. Colors identify risk zones, with purple indicating extreme ionized hypocalcemia.

Figure 5 displays a histogram for low ionized calcium critical limits. Color-coding reflects risk levels (green, low; yellow, medium; red, high; and purple, extremely high).

### 3.4. Blood Gas and pH Tests

Table 2 summarizes the POC blood gas and pH low and high critical limits for adults and newborns (in adult hospitals).

### 3.5. Hematology and Coagulation Tests

Table 3 summarizes the POC hematology and coagulation low and high critical limits for adults and newborns (in adult hospitals).

**Figure 6:**
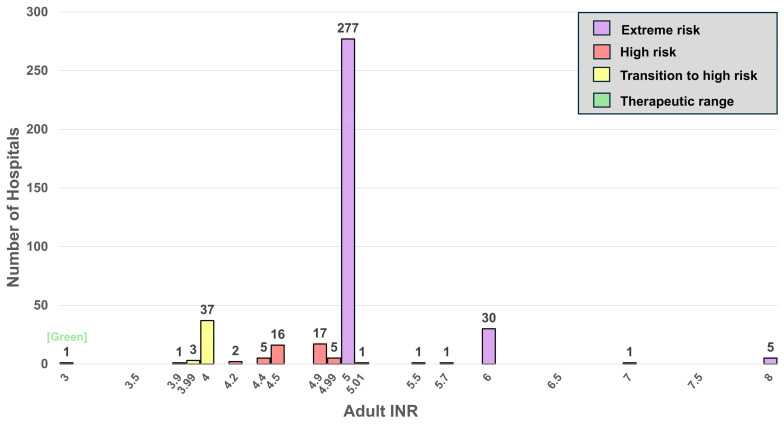
Histogram for high INR critical limits. Colors identify risk zones, with purple indicating extreme risk. The green zone can be considered safer, placing patients within the therapeutic range.

**Figure 7:**
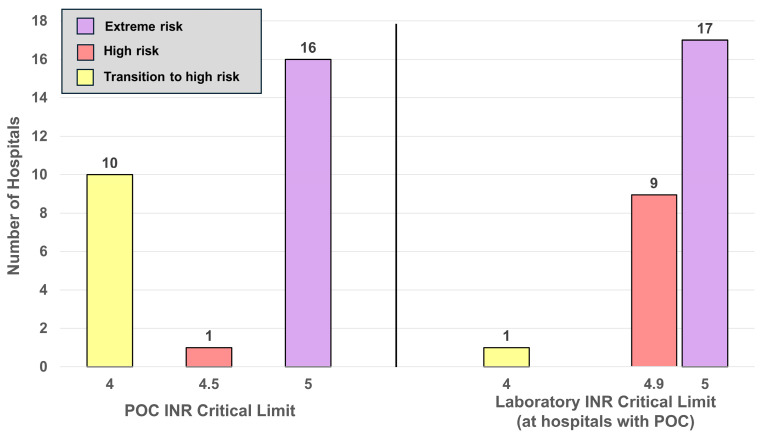
Histogram for high INR critical limits POC versus laboratory. POC versus laboratory adult INR matched pairs show no critical limits within the green zone, the therapeutic range. The middle black line separates the adult histograms for POC and laboratory INR. This figure includes critical limits only from hospitals that perform POC testing for INR.

For high INR critical limits, Figure 6 and Figure 7 display histograms for adults (*n* = 403) and POC versus laboratory adult-matched pairs (*n* = 27), respectively. Adult critical limits are widely distributed, with a range of 3–8 and a median of 5 INR (Figure 6). These figures are color-coded for risk zones (green, low; yellow, medium; red, high; and purple, extremely high). In Figure 7, laboratory and POC matched pairs range from 4 to 5 with a median of 5 INR for both.

### 3.6. POC Versus Laboratory Matched Pairs

Table 4 compares matched pairs of POC versus laboratory critical limits. The five analytes listed in the left-hand column have statistically significant paired differences. The effect sizes (rank-biserial correlations) for the five analytes are −1.000 for adult INR, 1.000 for low hemoglobin, −0.924 for low ionized calcium, 0.868 for high ionized calcium, and −1.000 for newborn INR.

Table 5 compares matched pairs of POC versus laboratory critical limits for adults and newborns at the same hospitals. For each measurand, the number of hospitals with matched POC and laboratory pairs is shown. This table also details relative differences in critical limits, that is, POC < Lab, POC = Lab, and POC > Lab.

A few measurands had identical critical limits for all POCT versus laboratory matched pairs, including: potassium (adult low and high, newborn low), sodium (adult and newborn low), CO_2_ content (adult high, newborn low), calcium (adult and newborn low), carboxyhemoglobin (adult and newborn high), methemoglobin (adult and newborn high), ionized calcium (newborn low), bilirubin (newborn high), pH (newborn low), arterial pCO_2_ (newborn high), and hematocrit (adult low). All laboratory values for newborn INRs were greater than POC.

## 4. Discussion

### 4.1. Glucose

Low and high glucose critical limits were the most frequently listed for point-of-care tests, reported by 73 hospitals (17.5%) for adults and 62 hospitals (14.9%) for newborns (in adult hospitals). Other measurands were listed less frequently. The ADA defines glucose <54 mg/dL (<3.0 mmol/L) as Level 2 hypoglycemia [6,7]. The majority of hospitals [69.9% (51/73)] listed adult hypoglycemic critical limits below 54 mg/dL (red and purple), 20.5% (15/73) notify at 54 mg/dL (yellow, associated with neuroglycopenic impairment), and 9.6% (7/73) fall in the timely notification zone (green). Only one hospital listed 70 mg/dL (Figure 3). The ADA designates the range from 54 to <70 mg/dL as Level 1 hypoglycemia. Eighteen hospitals listed hypoglycemic critical limits at or below 45 mg/dL (purple)—the extreme risk zone. Figure 4 shows that hyperglycemic critical limits are generally well above the range of 140 to 180 mg/dL (green) advised by the ADA for the maintenance of critically ill patients [7]. Both histograms (Figure 3 and Figure 4) show that notification thresholds are neither consistent nor aligned with clinician recommendations.

Critical limits should reflect evidence-based cutoffs and professional guidelines to mitigate patient risk and avoid adverse outcomes. Level 1 hypoglycemia (54 to <70 mg/dL) is the threshold for adrenergic responses to decreasing glucose levels in people without diabetes. Current 2026 ADA guidelines state that, “…a measured glucose level < 70 mg/dL (<3.9 mmol/L) is considered clinically important, regardless of symptoms.” Level 2 hypoglycemia (<54 mg/dL) is the threshold at which neuroglycopenic symptoms begin to occur; it requires immediate action to resolve the hypoglycemic event. Level 3 hypoglycemia is defined by the ADA as “a severe event characterized by altered mental and/or physical functioning that requires assistance from another person for recovery, irrespective of glucose level”. It can progress to loss of consciousness, seizure, coma, or death [6,7,8].

### 4.2. Troponin

Troponin, a highly specific and sensitive biomarker for cardiac injury, is essential for diagnosing acute coronary syndrome (ACS) and myocardial infarction [9,10]. In our national database of 417 hospitals, only 10 listed POC troponin, with notable variability in critical limits and the selection of cardiac biomarkers. Adopting high sensitivity tests and standardizing reporting practices could enhance the early diagnosis of acute myocardial infarction, streamline patient management, and improve outcomes [11,12].

### 4.3. Ionized Calcium

Severe ionized hypocalcemia is associated with high cardiac risk (hypotension, electromechanical dissociation, and cardiac output failure) [13,14]. Nearly half of the hospitals [47.6% (10/21)] list adult ionized hypocalcemia critical limits at 0.50 mmol/L (purple), a level which may produce tetany or life-threatening complications [15,16]; one hospital notifies at 0.75 mmol/L (red), a concentration (>0.5 to 0.75 mmol/L) that may require calcium administration [15,16]; while 42.9% (9/21) notify at 0.79–0.80 mmol/L (yellow), falling within the therapeutic range [17]. Only one hospital listed 1.00 mmol/L (green), following a more, perhaps overly conservative notification threshold. The histogram (Figure 5) highlights the need for harmonizations of critical notifications to enhance consistency and improve patient care.

### 4.4. INR

Thirty-seven percent of hospitals (10/27) notify at an INR of 4.0 (yellow), and 3.7% (1/27) at an INR of 4.5 (red). At INR ≥ 4.0, the odds ratio for intracranial hemorrhage (ICH) increases to approximately 8.8 compared with the therapeutic range of 2.0–3.0 [18,19]. Despite this, more than half of the hospitals [59.3% (16/27)] do not notify until an INR of 5.0 (purple), exposing patients to markedly elevated risk (Figure 7), as each 1-unit increase above an INR of 4.0 is associated with an approximate doubling of ICH risk [20,21].

Comparison of matched POC versus laboratory adult INR critical limits suggest that POC critical limits are more conservative (Figure 7 and Table 5). A similar pattern is observed for newborn INRs, in which laboratory critical limits exceed POC values in all cases (Table 5). Across all hospitals, adult INR critical limits show substantial variability, ranging from 3 to 8 (Figure 6). Notably, only one hospital [0.25% (1/403)] triggers notification at an INR of 3—within the therapeutic range—while most hospitals [78.4% (316/403)] list critical limits at INR ≥5.0, placing them in the extreme-risk category (purple) [20,21].

### 4.5. Hemoglobin

Nearly 70% of hospitals (16/23) list adult low hemoglobin critical limits below 7 g/dL, falling beneath the <7 g/dL threshold commonly used in transfusion practices [22,23]. Additionally, 21.7% (5/23) set notification thresholds below 6.5 g/dL, a level generally considered life-threatening because of the risk of severe tissue hypoxia and death [24,25]. This variability suggests a lack of consensus regarding hemoglobin thresholds that warrant urgent clinician notification or may reflect differing institutional risk tolerance, transfusion practices, or concerns about alert fatigue. Such heterogeneity has potential implications for patient safety and underscores the need for clearer guidance and consistency.

### 4.6. Comparison of POC and Laboratory Critical Limits

Table 4 compares POC and laboratory matched pairs. Three measurands demonstrate statistically significant differences—INR, ionized calcium and hemoglobin. In contrast, other analytes show no statistically significant differences in matched pairs, and some demonstrate identical critical limits, including potassium, sodium, CO_2_ content, calcium, carboxyhemoglobin, methemoglobin, ionized calcium, newborn bilirubin, pH, arterial pCO_2_, and hematocrit (Table 5). This likely reflects efforts to align POC testing with laboratory notification thresholds, consistent with quality management principles outlined in professional guidance [26] which emphasizes acceptable agreement and harmonization between POC and laboratory testing to support reliable clinical decision-making. In some cases, such as INR (Figure 7), the results suggest that POC critical limits are more conservative.

### 4.7. pH

While the adult median low pH critical limit was higher than the 7.0 pH threshold associated with significantly increased risk of mortality [27], a subset of hospitals continued to designate pH 7.0 as their critical limit (Table 2). This practice may delay provider notification until patients reach severe acidemia. Decreasing blood pH is associated with worsening neurologic impairment, including drowsiness, stupor, coma, and ultimately death [28]. As shown in Table 2, all hospitals set low pH critical limits below the 7.35 pH threshold defining acidemia and high pH critical limits above the 7.45 pH threshold defining alkalemia [29].

### 4.8. POC Adoption

Prior studies demonstrate that performing testing closer to the patient shortens time to urgent clinical interventions and may improve operational performance in emergent settings where time is critical [30]. From our national database of 417 hospitals, only 94 (22.5%) included point-of-care critical limits and values on their notification lists. Of the analytes, low and high glucose critical limits were the most frequently listed for point-of-care tests. These were reported by 73 hospitals for adults and 62 hospitals for newborns. Other measurands were listed less frequently. Inclusion of POCT critical limits in notification lists should be adopted by all hospitals to enhance patient safety and support timely clinical decision-making.

### 4.9. Limitations

Quantitative troponin decision thresholds could not be analyzed because of the extreme inconsistency in values and units used by hospitals. Please note that with the Wilcoxon signed-rank test, a significant difference may result from the distributions being dissimilar, even when the medians might be the same. Requests to confirm POC results using a second device were rarely encountered, which contrasts with recommendations from CLSI EP09 guidelines and some authors who emphasize verification using a central laboratory method rather than repeating the test on another POC platform when result accuracy is in question [31,32]. Another rare observation in notification lists was the specification of different critical limits for the same analyte across devices within the same hospital. Such discrepancies may lead to inconsistent clinical responses. Harmonization of critical decision thresholds is recommended to align alerts with evidence-based laboratory standards and clinical guidelines [33,34]. Reference ranges (normal subject intervals) were not captured in our national database; therefore, we were unable to statistically quantify deviations from low and high critical limits. We recommend that future research investigate these deviations, as they may serve as important metrics of unacceptable patient risk.

## 5. Conclusions

A major concern identified in this study is that several critical limits in use do not correspond to analyte concentrations linked to serious clinical outcomes. As illustrated in the histograms for glucose (Figure 3 and Figure 4), ionized calcium (Figure 5), and INR (Figure 6 and Figure 7), this discrepancy could delay detection of life-threatening conditions. These histograms demonstrate that few hospitals, if any (for example, Figure 7), notify within the timely notification zone (green), whereas most notifications fall within higher risk zones (yellow, red, and purple).

Uniform critical limit notification thresholds across POCT, central laboratories, and different devices could support more reliable clinical decision-making. Unlike central laboratories, which rely on communication loops that may introduce delays, POCT results are available immediately at the bedside, enabling prompt clinical action.

This dual immediacy of result availability and decision-making confers unique value to POCT for time-sensitive, life-saving interventions that central laboratory testing cannot match. Therefore, we recommend including POCT critical limits in notification lists to facilitate more rapid, potentially life-saving interventions.

Establishing standards, annual updating, and enforcing risk mitigation could enhance adoption and reliability. Publicly posting notification lists may help hospitals identify newly implemented tests and associated critical-decision thresholds, track and respond to changes in critical limits, and facilitate collaboration among medical centers toward greater harmonization of critical thresholds. This approach has the potential to promote more consistent and timely clinical responses and, ultimately, improve patient care and outcomes.

## Figures and Tables

**Table 1:** Critical limits for point-of-care clinical chemistry tests.

Measurand	Number of Hospitals (%)	Units	Low Mean (SD)	Low Median (Range)	High Mean (SD)	High Median (Range)
**1.A. Adults**						
Glucose	73 (17.5)	mg/dL	50.3 (4.9)	50 (40–70)	467.7 (60.5)	450 (200–600)
		mmol/L	2.8 (0.3)	2.8 (2.2–3.9)	26 (3.4)	25 (11.1–33.3)
Ionized calcium	21 (5)	mmol/L	0.66 (0.17)	0.75 (0.50–1.00)	1.65 (0.10)	1.60 (1.40–1.75)
Potassium	20 (4.8)	mmol/L	2.9 (0.2)	3 (2.5–3)	6.1 (0.2)	6 (6–6.5)
Sodium	18 (4.3)	mmol/L	123.6 (3)	126 (120–126)	156.7 (2.4)	155 (155–160)
CO_2_ content	17 (4.1)	mmol/L	10.7 (0.6)	11 (10–12)	40 (1.9)	39 (39–45)
Lactic Acid	14 (3.4)	mmol/L	…	…	3.6 (0.6)	3.9 (2–4)
Calcium	11 (2.6)	mg/dL	6.5 (0.2)	6.6 (6–6.6)	13 (0.3)	12.9 (12.9–14)
		mmol/L	1.64 (0.05)	1.65 (1.5–1.65)	3.25 (0.08)	3.23 (3.23–3.5)
Venous Lactic Acid	10 (2.4)	mmol/L	…	…	3.9 (0)	3.9 (3.9–3.9)
**1.B. Newborns**						
Glucose	62 (14.9)	mg/dL	44.7 (5)	45 (40–54)	227.7 (87)	200 (125–500)
		mmol/L	2.5 (0.3)	2.5 (2.2–3)	12.6 (4.8)	11.1 (6.9–27.8)
Ionized calcium	18 (4.3)	mmol/L	0.86 (0.05)	0.90 (0.80–0.90)	1.41 (0.14)	1.30 (1.30–1.60)
Potassium	15 (3.6)	mmol/L	3 (0.1)	3 (2.8–3)	6.8 (0.5)	7.1 (6–7.1)
Sodium	14 (3.4)	mmol/L	127.5 (4.3)	130 (120–130)	151.1 (2.9)	150 (150–160)
CO_2_ content	12 (2.9)	mmol/L	11 (0.4)	11 (10–12)	39.2 (0.4)	39 (39–40)
Calcium	11 (2.6)	mg/dL	6.9 (0.3)	7 (6–7)	13 (0.3)	12.9 (12.9–14)
		mmol/L	1.73 (0.08)	1.75 (1.5–1.75)	3.25 (0.08)	3.23 (3.23–3.5)
Bilirubin	10 (2.4)	mg/dL	…	…	17.9 (0)	17.9 (17.9–17.9)
		µmol/L	…	…	306.1 (0)	306.1 (306.1–306.1)

Abbreviation: SD, standard deviation. For units conversion, see Section 2.7.

**Table 2:** Critical limits for point-of-care blood gas and pH tests.

Measurand	Number of Hospitals (%)	Units	Low Mean (SD)	Low Median (Range)	High Mean (SD)	High Median (Range)
**2.A. Adults**						
pH	Low: 29 (7) High: 28 (6.7)	pH Units	7.20 (0.06)	7.20 (7.00–7.30)	7.59 (0.04)	7.60 (7.50–7.65)
Arterial pO_2_	Low: 27 (6.5) High: 1 (0.2)	mm Hg	45 (5.9)	40 (40–55)	200 (…)	200 (200–200)
		kPa	6 (0.8)	5.3 (5.3–7.3)	26.7 (…)	26.7 (26.7–26.7)
Arterial pCO_2_	Low: 20 (4.8) High: 21 (5)	mm Hg	20.3 (1.1)	20 (20–25)	67.1 (5.1)	70 (50–70)
		kPa	2.7 (0.1)	2.7 (2.7–3.3)	8.9 (0.7)	9.3 (6.7–9.3)
Carboxyhe- moglobin	21 (5)	%	…	…	12.6 (4.4)	10 (10–20)
Methemo- globin	20 (4.8)	%	…	…	4.5 (1.8)	4 (3–10)
Venous pH	20 (4.8)	pH Units	7.21 (0.02)	7.20 (7.20–7.30)	7.58 (0.05)	7.60 (7.50–7.65)
Venous pCO_2_	Low: 15 (3.6) High: 17 (4.1)	mm Hg	30 (7.3)	35 (20–35)	68.5 (4.1)	71 (56–71)
		kPa	4 (1)	4.7 (2.7–4.7)	9.1 (0.5)	9.5 (7.5–9.5)
Arterial O_2_ Saturation	11 (2.6)	%	76.8 (10.6)	80 (45–80)	…	…
Venous O_2_ Saturation	10 (2.4)	%	60 (0)	60 (60–60)	…	…
Arterial O_2_ Hemoglobin	10 (2.4)	%	79.5 (4)	82 (75–85)	…	…
Venous pO_2_	Low: 10 (2.4) High: 1 (0.2)	mm Hg	32 (0)	32 (32–32)	200 (…)	200 (200–200)
		kPa	4.3 (0)	4.3 (4.3–4.3)	26.7 (…)	26.7 (26.7–26.7)
**2.B. Newborns**						
pH	Low: 25 (6) High: 20 (4.8)	pH Units	7.19 (0.04)	7.20 (7.00–7.27)	7.59 (0.05)	7.60 (7.45–7.65)
Arterial pO_2_	Low: 23 (5.5) High: 1 (0.2)	mm Hg	45 (9.2)	40 (30–60)	200 (…)	200 (200–200)
		kPa	6 (1.2)	5.3 (4–8)	26.7 (…)	26.7 (26.7–26.7)
Arterial pCO_2_	Low: 17 (4.1) High: 22 (5.3)	mm Hg	23.8 (3.8)	25 (20–35)	70.9 (7.2)	70 (50.1–80)
		kPa	3.2 (0.5)	3.3 (2.7–4.7)	9.5 (1)	9.3 (6.7–10.7)
Carboxyhe- moglobin	20 (4.8)	%	…	…	11.5 (5.1)	9 (8–20)
Venous pH	19 (4.6)	pH Units	7.20 (0)	7.20 (7.20–7.20)	7.60 (0.04)	7.60 (7.55–7.65)
Venous pO_2_	Low: 10 (2.4) High: 1 (0.2)	mmHg	32 (0)	32 (32–32)	200 (…)	200 (200–200)
		kPa	4.3 (0)	4.3 (4.3–4.3)	26.7 (…)	26.7 (26.7–26.7)
Arterial O_2_ Saturation	10 (2.4)	%	80 (0)	80 (80–80)	…	…
Venous O_2_ Saturation	10 (2.4)	%	60 (0)	60 (60–60)	…	…
Methemo- globin	10 (2.4)	%	…	…	6 (1.5)	6 (5–10)
Venous pCO_2_	9 (2.2)	mmHg	22.8 (2.6)	25 (20–25)	62.2 (2.6)	60 (60–65)
		kPa	3 (0.4)	3.3 (2.7–3.3)	8.3 (0.4)	8 (8–8.7)
Capillary pH	Low: 9 (2.2) High: 4 (1)	pH Units	7.20 (0)	7.2 (7.20–7.20)	7.65 (0)	7.65 (7.65–7.65)
Capillary pCO_2_	Low: 4 (1) High: 9 (2.2)	mmHg	20 (0)	20 (20–20)	73.3 (7.9)	80 (65–80)
		kPa	2.7 (0)	2.7 (2.7–2.7)	9.8 (1.1)	10.7 (8.7–10.7)
Arterial O_2_ Hemoglobin	9 (2.2)	%	78.9 (3.7)	82 (75–82)	…	…

Abbreviation: SD, standard deviation.

**Table 3:** Critical limits for point-of-care hematology and coagulation tests.

Measurand	Number of Hospitals (%)	Units	Low Mean (SD)	Low Median (Range)	High Mean (SD)	High Median (Range)
**3.A. Adults**						
INR	27 (6.5)	INR	…	…	4.6 (0.5)	5 (4–5)
Hematocrit	Low: 24 (5.8) High: 12 (2.9)	%	19.7 (1.5)	20 (15–21)	61.3 (3.1)	60 (55–65)
Hemoglobin	Low: 23 (5.5) High: 6 (1.4)	g/dL	6.8 (0.7)	6.6 (6–8)	19.1 (0.4)	18.9 (18.9–20)
**3.B. Newborns**						
Hematocrit	Low: 21 (5) High: 10 (2.4)	%	25.3 (5.1)	25 (18–30)	68 (6.3)	65 (60–75)
Hemoglobin	Low: 18 (4.3) High: 7 (1.7)	g/dL	8.8 (1.5)	10 (6–10)	19.6 (1.5)	18.9 (18.9–23)
INR	12 (2.9)	INR	…	…	4.1 (0.3)	4 (4–5)

Abbreviation: SD, standard deviation.

**Table 4:** Statistically significant POC versus laboratory critical limit matched pairs.

Measurand, High or Low Critical Limit	Number of Hospitals	Units	*p*-Value	POC Median (Range)	Laboratory Median (Range)
**4.A. Adults**					
INR, High	27	INR	0.003	5 (4–5)	5 (4–5)
Hemoglobin, Low	23	g/dL	0.048	6.6 (6–8)	6.6 (6–7)
Ionized calcium, Low	21	mmol/L	0.002	0.75 (0.50–1.00)	0.76 (0.75–0.85)
Ionized calcium, High	21	mmol/L	0.004	1.60 (1.40–1.75)	1.59 (1.40–1.62)
**4.B. Newborns**					
INR, High	10	INR	0.003	4 (4–4.5)	4.9 (4.9–5)

**Table 5:** POC versus laboratory matched pairs qualitative difference.

Measurand	Number of Hospitals	POC < Laboratory		POC = Laboratory		POC > Laboratory	
		Low	High	Low	High	Low	High
**5.A. Clinical Chemistry**							
**Adults**							
Glucose	73	2	4	64	67	7	2
Ionized calcium	21	13	3	7	8	1	10
Potassium	20	…	…	20	…	…	…
Sodium	18	…	1	18	16	…	1
CO_2_ content	Low: 16 High: 15	…	…	15	15	1	…
Lactic Acid	14	…	…	…	13	…	1
Calcium	11	…	…	11	10	…	1
**Newborns**							
Glucose	62	3	3	52	56	7	3
Ionized calcium	17	…	…	17	16	…	1
Potassium	14	…	1	14	13	…	…
Sodium	12	…	1	12	11	…	…
CO_2_ content	12	…	…	12	11	…	1
Calcium	11	…	…	11	10	…	1
Newborn Bilirubin	10	…	…	…	10	…	…
**5.B. Blood Gas and pH**							
**Adults**							
pH	Low: 18 High:17	1	2	16	15	1	…
Arterial pO_2_	16	…	…	15	…	1	…
Carboxyhemoglobin	11	…	…	…	11	…	…
Arterial pCO_2_	10	…	1	…	9	…	…
Methemoglobin	10	…	…	…	10	…	…
Venous pH	10	…	1	9	9	1	…
**Newborns**							
pH	11	…	…	11	…	…	…
Arterial pO_2_	11	…	…	10	…	1	…
Arterial pCO_2_	11	…	…	11	…	…	…
Carboxyhemoglobin	10	…	…	…	10	…	…
Methemoglobin	10	…	…	…	10	…	…
**5.C. Hematology and Coagulation**							
**Adults**							
INR	27	…	10	…	17	…	…
Hemoglobin	23	…		18	…	5	…
Hematocrit	15	…	…	15	…	…	…
**Newborns**							
Hemoglobin	18	1	…	17	…	…	…
Hematocrit	13	1	…	12	…	…	…
INR	10	…	10	…	…	…	…

## Data Availability

The datasets presented in this article are not readily available due to confidentiality of sources and direct collection of many lists. Requests to access the datasets should be directed to geraldkost@gmail.com.

## Supplementary Materials

The following supporting information can be downloaded at: https://www.mdpi.com/article/10.3390/diagnostics16081139/s1; Table S1: POINT-OF-CARE CRITICAL LIMITS AND CRITICAL VALUE.

## Abbreviations

The following abbreviations are used in this manuscript:

ACS	Acute coronary syndrome
ADA	American Diabetes Association
AI	Artificial intelligence
CLSI	Clinical and Laboratory Standards Institute
COVID-19	Coronavirus infectious disease-2019
CTR	Center for Teaching and Research
ICH	Intracranial hemorrhage
INR	International normalized ratio
JASP	Jeffrey’s Amazing Statistics Program
MICU	Medical Intensive Care Unit
POC	Point-of-care
POCT	Point-of-care testing
TnI	Troponin I
