# Supplementary material for: Critical Decision Thresholds for Urgent Physician Notification of Point-of-Care Testing Results
Source: Diagnostics (Basel). 2026 Apr 10;16(8):1139. doi: 10.3390/diagnostics16081139 (PMC13115040; doi:10.3390/diagnostics16081139)
Supplement: Supplementary file 1 [file diagnostics-16-01139-s001.zip › diagnostics-4214191-supplementary.pdf]

Critical Decision Thresholds for Urgent Physician Notification of Point-of-care Testing Results

Supplement

by **Kami Osher [1] and Gerald J. Kost [2,*]**

TABLE S1. POINT-OF-CARE CRITICAL LIMITS AND CRITICAL VALUE

Measurand	Number of Hospitals (%)	Units	Low Mean (SD)	Low Median (Range)	High Mean (SD)	High Median (Range)
**A. Clinical Chemistry**						
**Adults**						
Creatinine	2 (0.5)	mg/dL	…	…	13.5 (2.1)	13.5 (12-15)
		μmol/L	…	…	1193.7 (187.6)	1193.7 (1061-1326.3)
Chloride	1 (0.2)	mmol/L	80 (…)	80 (80-80)	120 (…)	120 (120-120)
**Newborns**						
Creatinine	2 (0.5)	mg/dL	…	…	2.5 (0.7)	2.5 (2-3)
		μmol/L	…	…	221.1 (62.5)	221.1 (176.8-265.3)
**B. Blood Gas and pH**						
**Adults**						
Mixed Venous pH	6 (1.4)	pH Units	7.22 (0.04)	7.20 (7.20-7.30)	7.51 (0.02)	7.50 (7.50-7.55)
Capillary pCO_2	5 (1.2)	mmHg	20 (0)	20 (20-20)	65.8 (1.8)	65 (65-69)
		kPa	2.7 (0)	2.7 (2.7-2.7)	8.8 (0.2)	8.7 (8.7-9.2)
Capillary pH	4 (1)	pH Units	7.20 (0)	7.20 (7.20-7.20)	7.65 (0)	7.65 (7.65-7.65)
Capillary pO_2	Low: 4 (1) High: 1 (0.2)	mmHg	35 (0)	35 (35-35)	200 (…)	200 (200-200)
		kPa	4.7 (0)	4.7 (4.7-4.7)	26.7 (…)	26.7 (26.7-26.7)
Mixed Venous pCO_2	Low: 1 (0.2) High: 2 (0.5)	mmHg	20 (…)	20 (20-20)	62.5 (9.2)	62.5 (56-69)
		kPa	2.7 (…)	2.7 (2.7-2.7)	8.3 (1.2)	8.3 (7.5-9.2)
Mixed Venous O_2 Saturation	1 (0.2)	%	60 (…)	60 (60-60)	…	…

**Newborns**						
pH Cord Blood	Low: 6 (1.4)<br>High: 4 (1)	pH Units	7.13<br>(0.10)	7.20<br>(7.00-7.20)	7.65<br>(0)	7.65<br>(7.65-7.65)
Capillary pO_2	Low: 4 (1)<br>High: 5 (1.2)	mmHg	25<br>(0)	25<br>(25-25)	120<br>(44.7)	100<br>(100-200)
		kPa	3.3<br>(0)	3.3<br>(3.3-3.3)	16<br>(6)	13.3<br>(13.3-26.7)
Cord pCO_2	4<br>(1)	mmHg	20<br>(0)	20<br>(20-20)	65<br>(0)	65<br>(65-65)
		kPa	2.7<br>(0)	2.7<br>(2.7-2.7)	8.7<br>(0)	8.7<br>(8.7-8.7)
Base Excess	1<br>(0.2)	mmol/L	-11<br>(…)	-11<br>(-11- -11)	…	…
Cord Base Excess	1<br>(0.2)	mmol/L	-11<br>(…)	-11<br>(-11- -11)	…	…
**C. Hematology and Coagulation**						
**Adults**						
PT Screen	2<br>(0.5)	Sec	…	…	26<br>(0)	26<br>(26-26)
**D. Toxicology**						
**Adults**						
Lead Screen	1<br>(0.2)	μg/dL	…	…	45<br>(…)	45<br>(45-45)
**E. Urinalysis (qualitative critical value)**						
**Adults**						
Urine Ketones	1<br>(0.2)	3+ or 4+				
